# Understanding oncological and sexual function outcomes with gynaecological organ preserving cystectomy in women with bladder cancer; a systematic review

**DOI:** 10.1002/bco2.70053

**Published:** 2025-07-24

**Authors:** Rebecca Martin, Harriet Wylie, Charlotte Moss, Shaista Hafeez, Pardeep Kumar, Anne Marie Rafferty, Mieke Van Hemelrijck

**Affiliations:** ^1^ King's College London London UK; ^2^ The Royal Marsden NHS Foundation Trust London UK

**Keywords:** anterior exenteration, bladder cancer, cystectomy, organ sparing, sexual function, sexual recovery, sexual rehabilitation, urothelial cancer, women, womens health

## Abstract

**Introduction and Objectives:**

Cystectomy for bladder cancer (BC) in women involves removing gynaecological organs and the anterior vaginal wall, significantly impacting sexual function (SF). Gynaecological organ‐preserving cystectomy (GOPC) aims to minimise toxicity, but limited studies assess its impact. We reviewed existing evidence.

**Methods:**

A systematic review was conducted using Ovid (Medline, Embase, PsycINFO, CINAHL) and Cochrane Library. Studies assessing survival and SF outcomes of GOPC and SF outcomes of standard cystectomy were included.

**Results:**

Fourteen studies (1049 screened) reported on small cohorts (11–41 patients). Most GOPC patients had ≤T2b N0 M0 disease, while standard cystectomy patients had up to T4/N1. In the GOPC cohort median follow‐up was 36 months.

Over a 16–70 month period, Disease‐Free Survival in GOPC patients was 80–100%. Due to heterogeneity in Patient‐Reported Outcome Measures (PROMS), a narrative analysis was performed.

GOPC patients reported high levels of sexual activity, reduced dyspareunia and moderate‐to‐high satisfaction. While SF initially declined, recovery improved over time, with Female Sexual Function Index (FSFI) scores exceeding the 26.2 dysfunction threshold in two studies by 12 months.

In standard cystectomy, sexual dysfunction was common, with varying distress levels and inadequate patient counselling.

**Conclusions:**

Understanding the outcomes of GOPC is limited by study design and measurement variability, and meta‐analysis was not possible. In this narrative review, oncological outcomes in the GOPC group appears to have equivalent oncological outcomes to a standard radical cystectomy in carefully selected female patients. Sexual recovery outcomes in either complete or partial sexual organ preserving cystectomy appear to be better than a standard female radical cystectomy. Further prospective studies, particularly those involving nerve‐sparing surgery, are needed. Women undergoing either standard cystectomy and GOPC commonly experience sexual dysfunction, and there is a need to improve pre‐ and post‐operative counselling.

## 1 INTRODUCTION

Worldwide, bladder cancer is one of the top 10 most common cancers.^1^, ^2^ Bladder cancer (BCa) is more common in men than women; however, in women, it is often diagnosed later, and the disease can be more aggressive.^3^, ^4^, ^5^, ^6^ Therefore, the proportion of women presenting with muscle‐invasive disease and requiring radical treatment is higher.^4^, ^7^, ^8^ The treatments for BCa can directly or indirectly impact a person's sexual function.^9^

Cystectomy is the primary treatment for muscle‐invasive or high‐risk non‐muscle‐invasive disease. In women, this involves removal of the bladder along with the uterus, ovaries and anterior vaginal wall, regional lymph nodes and in many cases urethra, and with this denervation and devascularisation of the clitoris is common.^10^, ^11^ It is hypothesised that preserving these organs may improve physical functioning and hormonal regulation post‐operatively.

Modern surgical techniques coupled with increased awareness of retaining female sexual function aim to minimise the impact on the neurovascular bundles to preserve clitoral function and in some cases will include vaginal and/or uterine and/or neurovascular preservation.^10^, ^12^ However, until recently, national and European guidelines recommended the removal of the gynaecological organs in women, citing insufficient evidence to advocate an organ preserving procedure.^13^ Despite sparse evidence, in 2024, the European Association of Urology introduced guidance supporting gynaecological organ preserving cystectomy (GOPC) for women into their disease management guidelines.^14^

Sexual rehabilitation is increasingly recognised as an important part of cancer recovery and supportive care. It is directly associated with improvements in long‐term quality of life.^15^, ^16^ However, to date, female sexual function preservation and rehabilitation are better addressed in the more commonly occurring female cancers, such as gynaecological and breast,^17^, ^18^, ^19^ whilst remaining an area of unmet need in women with BCa.^20^, ^21^

We, therefore, conducted a systematic review of the literature to examine the role of gynaecological organ‐preserving cystectomy in women, its impact on sexual function and oncological efficacy.

The review question and aims used an iterative process in consultation with the authors, academic, clinical colleagues and patients. The review seeks to answer the following questions:

- Does gynaecological organ‐preserving cystectomy have oncological efficacy?
- How does a gynaecological organ‐preserving cystectomy impact sexual function?
- How does GOPC compare with a standard approach radical cystectomy regarding sexual function in women

## 2 METHODS

We followed the PRISMA guidelines for Systematic Reviews.^22^ This PROSPERO registered review aims to evaluate the available evidence on gynaecological organ‐preserving cystectomy for women with bladder cancer, focusing on oncological and sexual functional outcomes (PROSPERO id: CRD42023445391).

### 2.1 Search strategy and inclusion criteria

The research question, strategy and inclusion and exclusion criteria were developed in an iterative process with the authors, academic and clinical colleagues and patients and were developed prior to commencing the literature search in October 2023. The search was repeated in April, 2025 to ensure the most current evidence was included. Relevant studies were identified within Cochrane Library, EBSCO Host (CINAHL, Medline and PsychMed), PubMed and Ovid Gateway (Embase and Ovid) using the listed search terms. The search strategies (see Table 1) were developed using key terms and phrases consistent with bladder cancer, oncological outcomes and sexual health. A previous scoping review highlighted that studies in this area are limited; therefore, the search criteria were intentionally broad. Test searches were used to ensure the terms captured results answering the intended aims of the review.

**TABLE 1:** Search strategy.

1	2	3	4i	4ii
Bladder cancer*	Radical cystectomy	Female*	Sexual function	Survival
Transitional cell carcinoma	Anterior exenteration	Woman	Sexual dysfunction	Surgical margin*
Urinary bladder cancer*	Fertility‐sparing cystectomy		Sexual well‐being	Surgical outcome*
Transitional cell malignancy	Uterine sparing cystectomy		Sexual health	Cancer outcome*
Bladder tumour*	Sexual organ sparing cystectomy		Sexual problems	Oncological outcome*
Transitional cell tumours	Uterus‐sparing cystectomy		Sexual difficulty	Cancer Recurrence*
Bladder malignancy	Uterus‐preserving cystectomy		Quality of life	Disease‐free survival
Bladder neoplasm	Fertility‐preserving cystectomy		Distress	Recurrence‐free survival
Malignant tumour of the urinary bladder	NOT radiotherapy		Bother	NOT molecular
Urothelial cancer			Intimacy	
Urothelial malignancy				
Urothelial tumour				

All studies reporting on sexual function outcomes for female patients with bladder cancer undergoing a cystectomy were considered for inclusion. The inclusion and exclusion criteria are listed below.

### 2.2 Inclusion criteria

All original research studies that included female cystectomy and sexual function outcomes.

### 2.3 Exclusion criteria

Any studies not available in English and those that excluded a female bladder cancer population in the reporting. Any studies older than 10 years.

### 2.4 Data abstraction

**FIGURE 1:**
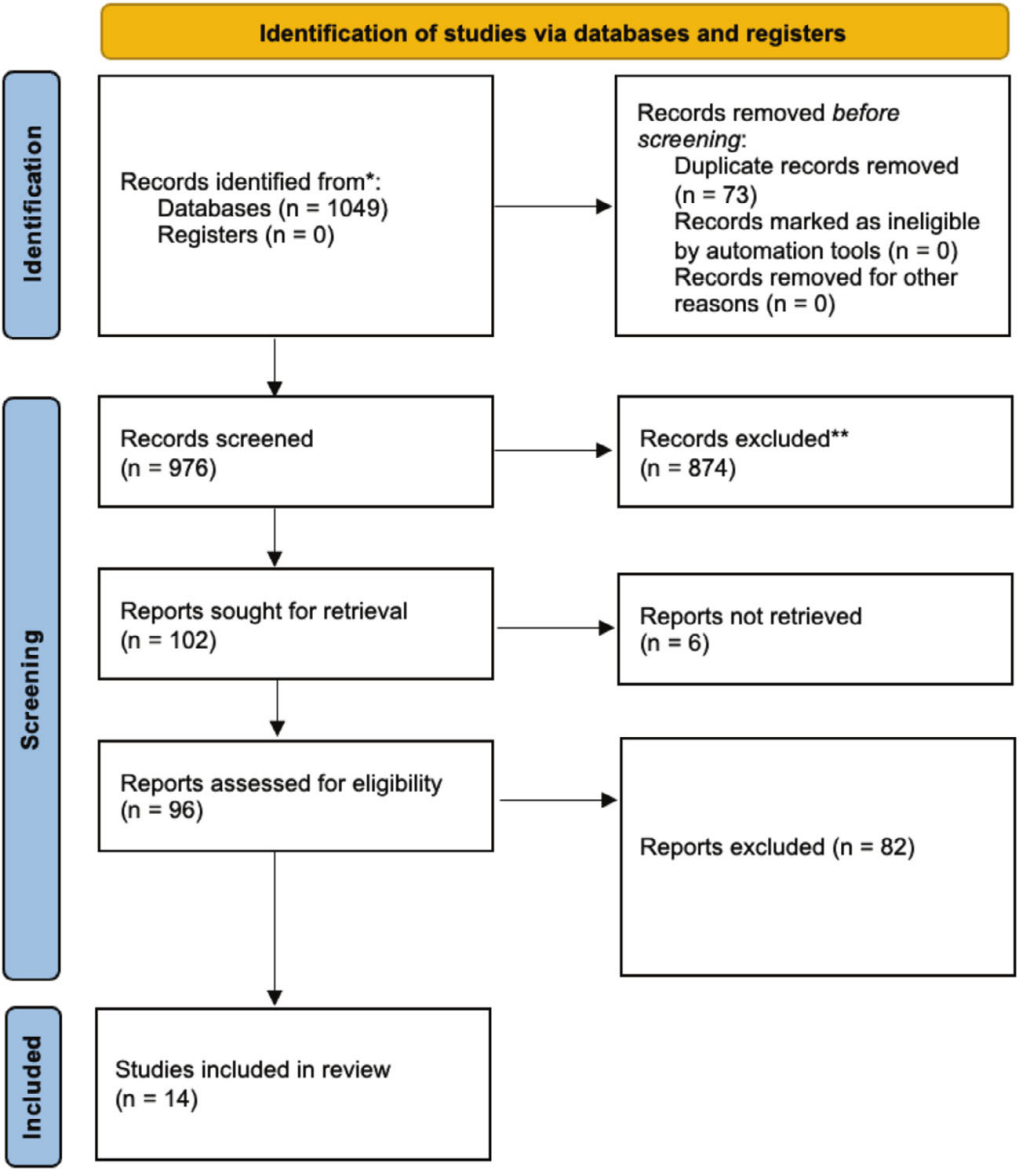
PRISMA diagram.

All duplicates were removed, and articles were reviewed initially by title, then abstract and finally full paper by two reviewers (RM and HW). Reviews, opinion articles and conference abstracts were excluded as part of the screening process (see Figure 1). References for each study were also reviewed to ensure no relevant citations were missed. Management of the screening process was done in Microsoft Excel.

All articles included in the final screening were quality assessed using the relevant tools provided by the Joanna Briggs Institute (JBI) criteria, and were excluded from the review if they failed to meet two or more of the criteria. One title was removed following this review. Final quality appraisals are included as part of Tables 2 and 3.

**TABLE 2:** Oncological outcomes.

Study no. (n = 14)	Author	Initial pathology/staging	Final pathology/staging	Type of study	No. pts.	Age	NAC	Sexual‐organ sparing surgery	ONB	FU (months)	DFS (%)	Positive margins	Time to recurrence (months)	Recurrence/type	Summary	JBI score
6	Cisternino et al, 2023	>/T3b N0 M0	Tis‐T3b N0 M0	Case series	14	57.6 (30–65)		100	100%	36	100				14 women followed up for 36 months. <T3 disease. Pre/post menopause 100% DFS.	10/10
11	Yang et al, 2022	>/T2	Tis‐ > T2	Case series	11	53	27%	100	100%	16	100				11 women followed up for 16 months. >/T2 disease. 100% DFS.	10/10
17	Lavellee et al, 2021	>/T2 N0 M0	Tis‐T2 N1	Case series	23	54 (34–79)	100%	100	87% ONB 13%IC	20	83	4%	13	Unknown	23 women followed up for 20 months. <T2 disease. 83% DFS. 4 patients developed recurrence at a median 13 months post‐op. 2 died of bladder cancer. 1 had a positive surgical margin (at ureter).	10/10
22	Tuderti et al, 2020	>/T2 N0 M0	Tis‐T3 N0	Case series	11	47.1	36%	100	100%	28	100	0%			11 women followed up for 28 months. <T3 disease. 100% DFS.	10/10
36	Moursy et al, 2016	>/T2b N0 M0	>/T2b N0	Case series	18	37.8 (32–43)		100	100%	67	100				18 women followed up for 28 months. <T2b disease. 100% DFS.	9/10
37	Roshdy et al, 2015	>/T2b N0 M0	T1‐T3 N1	Case series	24	51 (45–60)		100	100%	48	96		9	Pelvic relapse	24 women followed up for 48 months. <T2b disease. 96% DFS. 1 patient relapsed in the pelvis. This patient had higher than anticipated final pathology.	9/10
65	Ali‐El‐Dein et al, 2013	/>T2b N0 M0	T2 N0 M0‐T4 N2 M0	Case series	13	52		100	100%	70	80%	8%	7.3	Pelvis, Bone	13 women followed up for 70 months. <T4 disease. 80% DFS. 2 patients had higher than anticipated final pathology and both recurred within 3–4 months (bone and pelvis). A third patient recurred at 15 months in bone. 15 patients had genital sparing operation (and therefore mean age/staging based on these numbers).	10/10

**TABLE 3:** Sexual function outcomes.

Study no. (n = 14)	Author	Final pathology/staging	Type of study	Age	No. patients	PROMS used	Sexual organ‐sparing	FSFI score (pre)	FSFI score (post)	FSFI score (post)	Pre‐op sexually active	Post‐op sexually active	Sexual interest/desire	Arousal	Pain	Lubrication	Orgasm	Enjoyment/satisfaction	Successful intromission	Body image	Intimacy concerns	Sexual wellbeing score	Bother/distress	Wish to retain SF	Summary	PISQ‐IR	JBI assessment
5	Wenk et al, 2023	T1‐T2	QoL	65.89	35	ICIQ‐VS pt a PISQ PISQ‐IR QLQ‐C30 BLM30	17% (Vaginal)			PISQ‐IR		34%													35 women. Mean age 65.89 years. 17% vaginal sparing cystectomy. PISQ‐IR survey delivered >6 months post op. 34% women sexually active post‐op. Reasons for sexual inactivity related to condition, global quality and condition impact. In a small number it was partner related. Scores in the vaginal sparing group were better than the non‐sparing group.	SA = 3.79 NSA‐PR = 2.5 NSA‐CS = 2.0 NSA‐GQ = 2.0 NSA‐CI = 1.33	10/10
6	Cisternino et al, 2023	Tis‐T3b N0 M0	Case series	57.6 (30–65)	14	FSFI	100%		18.3 (at 3 months)	29.1 (12 months)	100%	100%	100%	85.50%	7%	78.50%	85.50%	85.50%							14 women all having sexual organ sparing cystectomy. FSFI administered at 3 and 12 month post‐op. Low score on FSFI at 3 months post op. Normal sexual function on FSFI at 12 month post op. Non‐standardised reporting of the individual domains is hard to interpret.		10/10
11	Yang et al, 2022	Tis‐ > T2	Case series	53	11	FSFI	100%	20.9	17.3 (at 6 months)		100%		2.4	2.7	3.2	3.3	2.8	2.8							FSFI measured pre op and 6 months post op. Reduction in FSFI score at 6 months post‐op.		10/10
13	Lofgren et al, 2022		QoL	64 59–73 (range)	10	Qualitative interviews	10%						C	C	C		C	C			C			Most/All	10 women interviewed. 10% with sexual organ sparing cystectomy. Overall theme: Balance emotional and physical closeness. Sensual relationship and Sexual relationship. 5 sub‐themes: Feeling of intimacy, The importance of the relationship, Her reluctance to engage in sexual activity, Partner's inability to engage in sexual activity and Acting for sexual rehabilitation. Surgery created sexual anxiety and reluctance to resume intercourse despite the desire to do so. Counselling is lacking.	C=Concern	9/10
17	Lavellee et al, 2021	Tis‐T2 N1	Case series	54 (34–79)	23	Unknown questionnaire	100%					87%	93%		10%		100%	38% high satisfaction 54%moderate satisfaction 8% low satisfaction							23 women all with sexual organ sparing cystectomy. SF measured using an unknown questionnaire at an unknown post‐op time point. The questionnaire had scores for each domain between 1 and 10 and were considered to represent high/moderate/low impact for each domain. High levels of sexual activity and satisfaction, low levels of dyspareunia. Of the 23 women, only 15 answered the sexual function survey, and some questions i.e. relating to body image only related to neobloadder		10/10
22	Tuderti et al, 2020	Tis‐T3 N0	Case series	47.1	11	QLQ‐30 BLM‐30 FSFI	100%	31.9	20.3 (3 months)	26.2 (at 12 months)		72.70%		4.2	5.2	5.1	4.4	4							11 women all with sexual organ sparing cystectomy. 72.7% sexually active post op. FSFI measure at baseline, 3 months and 12 months. FSFI scores had a decline at 3 months and return to within normal range by 12 months, although not to pre‐operative levels in all domains.		10/10
36	Moursy et al, 2016	>/T2b N0	Case series	37.8 (32–43)	18	Self reported	100%				100%	100%			16%										18 women all with sexual organ sparing cystectomy. SF measured post‐op through self‐reporting about pain and orgasm at an unknown time point. All reported sexual activity during the follow up period and ‘normal’ orgasms. 18% of patients reported dyspareunia. This is not relevant as such as not looking at oncological outcomes but vast majority squamous cell… they do report reoccurence rates, is squamous cell more/less aggressive than TCC? Also are all these patients pre‐menopausal? Young mean age		10/10
37	Roshdy et al, 2015	T1‐T3 N1	Case series	51	24	Modified IFSF Self reported	100%					92%		91%	14%	91%	86%		100%						24 women all with sexual organ sparing cystectomy. Only 22 women reported functional outcomes. SF measured using a modified FSFI at an unknown post‐op time point and with non‐standardised reporting. Results hard to interpret but appears to be 14% of patients had some dyspareunia. 92% reported sexual activity post‐op. 22/22 women able to achieve vaginal intercourse		10/10
52	Booth, Rasmussen & Jensen, 2015		Cross section	67	41	FSFI and Unknown Questionnaire	0 (complete)12% vaginal sparing			4.8 (median)	78%	37%	1.5	0.9	0	0	0	3.2		11% altered body image affecting sex life Reduced femininity					Overall reduction sexual activity 54% decreased desire post 44% harder to orgasm. Broad range of scores relating to questions on satisfaction with sex life and emotional status relating to sexuality from not bothered to greatly bothered. 30% patients wanted more info on sexual consequences/rehab. Pre‐op/post op. 37% reduced sensitivity. Questionnaire was adminstered between 1 and 4 years post‐op		8/8
57	Westerman et al, 2021	Tis‐T4	QoL	61.7	22	Bespoke questionnaire	0 (complete) 72% vaginal sparing (partial/complete)				81.80%		C		60%	C		C 15% altered sensation	C 20% unable				Fear of penetration due to pain		22 women all post cystectomy. All women reported at least 1 change in SF post‐op, most commonly dyspareunia (60%). 22% unable to have ‘sexual activity’. Only 46% received pre‐op counselling on SF consequences, a further 27.3% received a little but desired more. Most women (77%) rated Vaginal sparing important. Only 31% received provider SF counselling post surgery. 77.8% wanted provider led counselling pre‐op.	C=Concern	8/8
59	Gupta et al, 2020	Tis‐T4	QoL	pre‐op 69 (median) post op 67.5 (median)	6 pre‐op cohort 16 post‐op cohort	Qualitative pre and post op cohorts)	81% vaginal sparing				67% pre‐op cohort	19% post op cohort		C	C			C		C	C		C	Y	Themes: Preop concerns: Body image‐concerns about the urostomy bag and body image. Worried about starting new relationships. Psychological health‐anxiety and need for greater provider led support. SF and intimacy‐concerns about SF post op and changes. Others more concerned about oncological outcomes. Post op: Body image‐altered wardrobe. Concerns with self confidence. Worried about odour and leakage. Altered sense of femininity. Psychological health‐all made a psychological adjustment, no lasting impact on psychological health. Feelings of isolation as a female BC patient. Need female peer connections. SF and intimacy‐concerns about new relationships. Altered desire. Dyspareunia inhibiting intercourse. For others, this was less significant. Different priotiy given to sexual recovery by participants. Both cohorts reported provider led SF counselling was lacking. Support groups were male dominated.		9/10
65	Ali‐El‐Dein et al, 2013	T2 N0 M0‐T4 N2 M0	Case series	52	13	FSFI UDS	100%			18		100%	2.5	3.5	3.2	2.8	3.4	2.9							13 women post sexual organ sparing cystectomy compared to 110 women standard cystectomy. SF measured using FSFI at an unknown time point. Sexual recovery was at 6 weeks, shorter than the standard cystectomy group at 14 weeks. With overall FSFI scores favourable to the standard cystectomy cohort, although scores in both groups are low 18 & 15 respectively.		10/10
256	Milling et al, 2024	Up to M1	QoL	72	151	Modified FSFI SHQ22	Unknown					23%													Resons for sexual inactivity: 31% lack of desire 35% pain 8% no partner 35% unknown SHQ22 Altered vaginal size and pain significant factors. Items including communication with HCP recorded high scores. 70% of women stated that surgery had affected their sexual activity.		10/10
257	Ceasar et al, 2024	Tis‐T4	QoL	70	40	Qualitative interviews	~40%																		4 key themes: 1. Receiving limited provider feedback about the impact of radical cystectomy on sexualfunction. 2. Wanting to engage in sex after surgery, but facing physical and mental barriers. 3. Feeling a lack of sexual arousal or climax post surgery. 4. What has been helpful: physical therapy to recover.		8/8

The following information was collected from each study at the full text stage: type, grade and stage of bladder cancer, mean age, extent of gynaecological organ preserving surgery including full, partial and nerve sparing, oncological outcomes including initial and final pathology, disease free survival and recurrence free survival, surgical margins, concurrent lymph node dissection, neo‐adjuvant and adjuvant treatment, type of urinary diversion. Sexual function data included information on pre‐treatment and post‐treatment counselling/PROMS, PROMS used, sexual activity, type and proportion of sexual dysfunction, sexual interest, sexual enjoyment, intimacy concerns, extent of distress, other themes.

RM performed the data abstraction, with HW verifying it. Any discrepancies in data abstraction or quality were resolved through discussion.

## 3 RESULTS

A total of 1049 references were identified through the search strategy. After title screening, 338 were included for abstract review and 102 references for full text review. Of those, 25 references were considered for full text analysis. After the full text analysis and removal of duplicates, 14 references met all inclusion criteria; 7 for survival and sexual function analysis and 7 for sexual function analysis alone (see Figure 1). See Tables 2 and 3 for a complete list of included texts.

The search produced a variety of studies that are broad in their methodology and findings; therefore, there was no scope for a meta‐analysis.^23^, ^24^ A Systematic Review Without Meta‐analysis (SWiM analysis) and narrative review have been performed.^23^, ^25^ Emerging findings and themes from each category are brought together and analysed in the discussion.

A broader selection of studies was included in the sexual function analysis, including several qualitative studies (n = 6) as well as cross‐sectional studies (n = 1). The sites of the studies were the USA (n = 3), Egypt (n = 2), Sweden (n = 2), Italy (n = 2) and Germany (n = 1), China (n = 1), Denmark (n = 2) and India (n = 1).

### 3.1 Gynaecological organ‐preserving cystectomy survival outcomes

Seven case series studies, each including 11–24 patients, were analysed (Table 2). Only studies reporting both survival and sexual function outcomes were included. All patients underwent complete gynaecological organ‐preserving surgery, with most receiving an orthotopic neobladder and one study including both ileal conduit and orthotopic neobladder cases. None described a nerve‐sparing approach, although an assumption could be made that full gynaecological organ preservation is nerve‐sparing due to the anatomical planes. Reporting on the inclusion of neo‐adjuvant chemotherapy nor delays to cystectomy in the patient pathway were not consistently recorded in the papers, thus limiting data analysis.

Patient selection for GOPC was based on radiological staging, absence of disease in key areas (bladder neck, trigone, urethra), negative gynaecological exams and pathology up to T3b N0. However, several cases showed more advanced final pathology than anticipated pre‐operatively.

Follow‐up ranged from 16 to 70 months at 3–6 month intervals, with imaging and functional outcome reviews. Positive margins were reported in two patients. Disease‐free survival (DFS) ranged from 80% to 100%, with no cases of relapse in gynaecological organs.

DFS of 100% was noted in four studies, with final pathology up to T3b N0 and follow‐up of 16–67 months. The remaining three studies reported recurrences in the pelvis and bones, with time to recurrence between 7.3 and 13 months. One study did not specify the recurrence type. With overall disease‐free survival across the seven studies at 80–100%.

### 3.2 Sexual function outcomes

An additional seven studies are included for sexual function analysis in the absence of survival outcomes. Table 3 presents the findings across all 14 papers. These studies included both GOPC and standard radical cystectomy cohorts, with sexual function being reported within all of them. In the GOPC cohort a key inclusion criterion in most studies was pre‐operative sexual activity and a motivation to retain sexual function post‐operatively.

Pre‐surgery sexual activity was not always specified in the studies. Post‐operatively, sexual activity was high in the GOPC cohort (72.7–100%, avg. 96%), although one study did not clarify this.^26^, ^27^, ^28^, ^29^, ^30^, ^31^, ^32^

Sexual activity and function were assessed using various methods, most commonly the Female Sexual Function Index (FSFI). However, FSFI usage lacked consistency; −only two studies recorded baseline scores, −of these, only one tracked FSFI over multiple follow‐ups^26^, ^28^, ^32^ ‐two studies reported FSFI non‐standardly, limiting parameter interpretation; author clarifications were requested but not received.^26^, ^30^

The two studies, both in the GOPC cohort, with pre‐ and post‐operative FSFI data, showed an initial decline of 8–17%, with recovery by 12 months, exceeding the 26.2 threshold for normal function.^26^, ^28^ One study reported initially low post‐operative FSFI scores (20.9), further declining at six months (17.3).^32^

One study reported on a cystectomy cohort with 17% of patients having vaginal sparing cystectomy, in this group sexual function scores were higher. However, in this study, sexual activity across all cohorts was reported to be reduced.^33^ The PISQ‐IR survey was used for evaluation (>6 months post‐op) and showed only 34% were sexually active, with inactivity linked to health, quality of life and partner issues.

Eight studies used alternative methods or a combination of alternative methods and the FSFI: ‐two self‐reported symptoms, −three used bespoke or unknown questionnaires and ‐three used qualitative interviews. Themes across the studies include: reduced sexual activity and sexual enjoyment in the post‐operative setting, reduced desire, sensitivity and orgasmic issues, dyspareunia, emotional and intimacy concerns, reduced self‐esteem and concerns about quality of counselling and support for women.^27^, ^29^, ^34^, ^35^, ^36^, ^37^, ^38^, ^39^ One study examined reasons for sexual inactivity, identifying several key factors; including anxiety about intimacy and vaginal intercourse, with specific anxiety about vaginal size and dyspareunia. Additional factors cited were anxiety about delayed orgasm or anorgasmia.^35^

## 4 DISCUSSION

Due to a heterogeneity in the reporting methods for both survival outcomes and sexual function outcomes, statistical analysis was not possible. In the absence of this direct comparison and statistical evaluation, this narrative review has found that gynaecological organ sparing cystectomy appears to have oncological outcomes similar to that of a standard approach anterior exenteration. The GOPC cohort appears to have high levels of sexual activity and some evidence of recovery of normal sexual function scores by 12 months post‐operatively.

Overall, there appears to be a negative correlation between cystectomy for women and sexual function. With reports of significant symptom burden.

### 4.1 Gynaecological organ‐preserving cystectomy survival outcomes

Although statistical analysis was not feasible due to a lack of homogeneity, survival outcomes in the GOPC papers appear to be similar to the standard of care, anterior exenteration. In the small number of papers included here with disease‐free survival reported as 80–100% at follow‐up periods up to 70 months. There appears to be improved sexual function outcomes in the GOPC cohort.

Accepted survival data suggest a 50% disease‐free survival at 60 months after cystectomy.^40^ However, many factors impact survival outcomes, including accurate staging and diagnosis, in conjunction with timely and appropriate treatment pathways. Notably, many papers within this review either did not include or did not report on the use of neo‐adjuvant chemotherapy (NAC). NAC is considered standard of care in European guidelines and has an associated 6% improved survival outcomes for patients with muscle‐invasive bladder cancer at 10 years.^40^, ^41^ In their 2020 paper, Russell et al reported that a delay in radical cystectomy after diagnosis was found to have a significantly detrimental effect on overall survival for bladder cancer patients.^42^ In the papers reported in this review, careful staging and patient selection are described. However, two of the papers in this review report staging as unexpectedly higher than anticipated. As neither delays to cystectomy nor standardised reporting of NAC are included in the papers, full survival analysis has not been possible.

Notably, in this review, none of the papers reported recurrence within the retained pelvic organs. Rather, the patterns of recurrence were consistent with expected patterns of recurrence in standard of care patients, ie, within the pelvic nodes or distant metastases.^40^ Huang et al reviewed 112 cases of female cystectomy at their institution comparing concurrent hysterectomy and uterine sparing cystectomy. They found low rates of uterine invasion and concluded that hysterectomy was not an independent predictor of survival outcomes.^43^ This is consistent with other studies evaluating gynaecological organ outcomes in female cystectomy patients. Vakarakis et al reported a low incidence of unsuspected gynaecological organ involvement in their retrospective case review, identifying such involvement in only three patients (5.7%). Specifically, the vagina was affected in two cases (3.8%), and the uterus in one case (1.9%).^44^ Vakarakis et al concluded there were no identified predictable features. A further recent study by Avulova et al found that in women with pT4 disease, the most involved pelvic organ is the anterior vaginal wall and therefore, routine removal of all pelvic organs may not be required.^45^

However, Choi et al, found that predictable factors for gynaecological organ involvement prior to cystectomy include tumour location at the trigone or bladder neck, a maximum tumour size at CT > 4.8 cm or hydronephrosis at CT as significant predictors of gynaecological organ involvement and should aid patient selection for uterine paring cystectomy.^46^ This is consistent with the findings by Gregg et al, in the largest case series evaluating gynaecological outcomes in women having cystectomy.^47^ Gregg et al found that a lack of trigonal/bladder floor tumour, palpable posterior mass and clinical lymphadenopathy is associated with the absence of pelvic organ involvement. Concluding that an individualised risk assessment using these factors, along with patient preferences, should be used to guide surgical planning.^47^ Consideration to preservation of the urethra should also be made if an orthotopic reconstruction is proposed, and this technique is well established with low rates of urethral recurrence.^48^ Indeed, the papers in this review each describe careful patient selection consistent with recommended practice.

### 4.2 Sexual function outcomes

In this review, sexual function outcomes appear improved in patients having complete GOPC, with resumption of sexual activity and lower symptom burden. Traditional cystectomy techniques involve partial resection of the anterior vaginal wall, and aside from the psychological and emotional factors associated with this, physical vaginal complications can include vaginal prolapse, neobladder vaginal fistulation and sexual dysfunction.^49^ Anatomical considerations are often focussed on vaginal length, perhaps with the assumption of penetrative intercourse. However, removal of the urethra and/or anterior vaginal wall impacts neurovascular innervation of the clitoris.^50^ A standard radical cystectomy will often include a lymph node dissection; the lymph node dissection surgical plane may impact clitoral nerve innervation. No papers were found that had investigated this approach. The clitoris is the primary organ for female sexual pleasure^51^ and techniques to preserve clitoral function may improve outcomes of sexual function and sexual satisfaction.

The measurement of sexual function across the included studies was inconsistent, with limited use of standardised tools, thereby presenting challenges for comparative analysis. The application of validated PROMs varied considerably. Only two studies collected baseline measures of sexual function, while two others applied a PROM at postoperative intervals (i.e. 3 and 12 months). One study administered a PROM at a single postoperative time point (6 months). Notably, only one study used a standardised PROM at three intervals: preoperatively, and at 3 and 12 months postoperatively.

A lack of standardised reporting methods limited clarity, interpretation and comparability of outcomes. These findings are consistent with other reviews, attempting to identify and analyse similar outcomes.^9^, ^21^, ^52^ Bessa et al. concluded that more studies were needed to develop, standardise and implement the use of sexual health questionnaires in clinical practice.^53^ The use of standardised units of measure is important for research to ensure validity, reliability and comparability of results.^54^

The effects of a cancer diagnosis on sexual wellbeing are well documented, along with the importance of maintaining sexual intimacy.^55^ This is reflected in this review, with women expressing the preference of retaining the option for sexual intimacy.^37^ This is, however, poorly captured in the standardised measures. Female sexual dysfunction is complex and impacted not just by physical, anatomical changes but also psychosexual factors.^56^ The commonly used PROMS do not necessarily reflect the complexity of female sexual dysfunction.^57^ For example, the FSFI, measures predominantly physical symptoms and has been criticised for both its ability to capture psychometric values in a cancer population and a heteronormative approach to female sexual function.^58^, ^59^ The existing QOL tools used in many studies have very limited measures on female sexual function centred around sexual activity and vaginal dryness and without consideration for the complex issues associated with FSD, disease specific concerns nor the psychometric associations of FSD.^17^, ^53^, ^56^ The EORTC Sexual Health questionnaire recently validated for psychometric values may address this gap.^60^ However, when examining surgery outcomes, application before and at regular intervals after will benefit understanding of these outcomes.

In this review, it seems that recovery of sexual function demonstrated improvement at 12 months after surgery in the GOPC cohort. There have been few longitudinal studies examining sexual recovery in bladder cancer patients, and fewer examining female sexual recovery, with many studies following a retrospective design. In prostate cancer, there is a widely accepted 2 year timeframe for recovery of spontaneous erections, although recovery can continue well beyond this timeframe.^61^ An extended timeframe for measuring outcomes would provide important longitudinal data, especially in the context of female sexual recovery.

There are clear risks of sexual dysfunction associated with female cystectomy. Therefore, all patients should be offered counselling both in the pre‐operative setting on risks and in the post‐operative setting on recovery. In this review, significant gaps were identified in the counselling of patients as well as gaps in peer support. Recent papers by Voigt et al and Davis et al offer that there an inherent gender bias within urological care that perhaps contributes to this gap within the service.^21^, ^62^

This review concurs with the conclusion of recent studies by Gupta et al and Vencill et al, that female sexual dysfunction within bladder cancer is poorly understood.^36^, ^63^ Several papers have made recommendations on ways to manage this in practice, however, this will be challenging to implement without a motivation for culture change and a focus on training.^21^, ^37^, ^64^, ^65^ Although the culture is changing across cancer care, multiple barriers have been identified to explain this phenomenon^17^, ^19^, ^63^, ^65^; −lack of knowledge or confidence on behalf of the treating clinician and ‐patient factors including the lack of confidence or validation to raise concerns, along with ‐absence of training on female sexual issues within urological training programmes.^17^, ^19^ This may lead to a cycle of problems being both under‐reported and under‐addressed.

### 4.3 Limitations

Due to heterogeneity in the reporting outcomes for both the disease‐free survival data and patient‐reported outcomes and measures, this review is limited in its reporting. A narrative review may be limited by a degree of reporting bias, and effort has been made through the process to minimise this. Two reviewers independently completed the review process, and discrepancies were resolved through team discussion, however, some subjectivity in interpretation may prevail.

Furthermore, the reporting of sexual function outcomes within the GOPC cohort is likely to be affected by patient selection bias. As a being sexually active and motivated to retain sexual function in most studies was needed to meet inclusion criteria for consideration of GOPC.

Effort was made to capture the most recent evidence, however, this is an emerging area of research, and the most recent studies may not have been included.

## 5 CONCLUSIONS AND RECOMMENDATIONS

Gynaecological organ preserving cystectomy in this narrative review, appears to have equivalent oncological outcomes to a standard radical cystectomy in carefully selected female patients. Sexual recovery outcomes in either complete or partial sexual organ preserving cystectomy appear to be better than a standard female radical cystectomy. In this gynaecological organ sparing group, sexual recovery improves over time, at 12 months following surgery, there appear to be high levels of sexual activity, sexual satisfaction and minimal dyspareunia. Despite being associated with improved quality of life outcomes, functional outcomes for women with cystectomy remains significantly under‐investigated. Further prospective comparative studies are required to investigate gynaecological organ preserving cystectomy in women are needed as well as investigation into nerve sparing approaches.

Furthermore, most patients having cystectomy will experience a degree of sexual dysfunction, the duration and extent are challenging to anticipate. Therefore, patient counselling on risks and recovery is fundamental, but remains lacking within clinical practice. Further studies are required to better understand and address this area of unmet need.

## AUTHOR CONTRIBUTIONS

*Access to all the data for this review and full responsibility for data integrity and accuracy of analysis*: Rebecca Martin. *Conception and design*: All authors. *Data acquisition*: Rebecca Martin, Harriet White and Charlotte Moss. *Analysis and interpretation*: Rebecca Martin and Harriet White. *Drafting of the manuscript*: Rebecca Martin. *Critical revision of the manuscript*: All authors.

## CONFLICT OF INTEREST STATEMENT

There are no conflicts to declare.
